# Spinal Motion and Muscle Activity during Active Trunk Movements – Comparing Sheep and Humans Adopting Upright and Quadrupedal Postures

**DOI:** 10.1371/journal.pone.0146362

**Published:** 2016-01-07

**Authors:** Stephanie Valentin, Theresia F. Licka

**Affiliations:** 1 Equine Clinic, University of Veterinary Medicine Vienna, Vienna, Austria; 2 Large Animal Hospital, Royal (Dick) School of Veterinary Studies, University of Edinburgh, Roslin, Scotland, United Kingdom; Semmelweis University, HUNGARY

## Abstract

Sheep are used as models for the human spine, yet comparative in vivo data necessary for validation is limited. The purpose of this study was therefore to compare spinal motion and trunk muscle activity during active trunk movements in sheep and humans. Three-dimensional kinematic data as well as surface electromyography (sEMG) of spinal flexion and extension was compared in twenty-four humans in upright (UR) and 4-point kneeling (KN) postures and in 17 Austrian mountain sheep. Kinematic markers were attached over the sacrum, posterior iliac spines, and spinous and transverse processes of T5, T8, T11, L2 and L5 in humans and over the sacrum, tuber sacrale, T5, T8, T12, L3 and L7 in sheep. The activity of erector spinae (ES), rectus abdominis (RA), obliquus externus (OE), and obliquus internus (OI) were collected. Maximum sEMG (MOE) was identified for each muscle and trial, and reported as a percentage (MOE%) of the overall maximally observed sEMG from all trials. Spinal range of motion was significantly smaller in sheep compared to humans (UR / KN) during flexion (sheep: 6–11°; humans 12–34°) and extension (sheep: 4°; humans: 11–17°). During extension, MOE% of ES was greater in sheep (median: 77.37%) than UR humans (24.89%), and MOE% of OE and OI was greater in sheep (OE 76.20%; OI 67.31%) than KN humans (OE 21.45%; OI 19.34%), while MOE% of RA was lower in sheep (21.71%) than UR humans (82.69%). During flexion, MOE% of RA was greater in sheep (83.09%) than humans (KN 47.42%; UR 41.38%), and MOE% of ES in sheep (45.73%) was greater than KN humans (14.45%), but smaller than UR humans (72.36%). The differences in human and sheep spinal motion and muscle activity suggest that caution is warranted when ovine data are used to infer human spine biomechanics.

## Introduction

Sheep are a commonly used animal model for the human spine, as anatomical similarities in the human and sheep thoracolumbar spine have been described [1]. In addition, the craniocaudal variation in range of motion of single-joint spinal segments under loaded conditions is similar between these species [2] and as a result, sheep are deemed particularly suitable for in vitro spinal range of motion testing [3]. Such in vitro studies are generally performed on functional spinal units devoid of soft tissue. Although in vitro investigations allow the possibility of measuring parameters which would be difficult or impossible to measure in vivo, the removal of soft tissue structures in vitro will alter the biomechanical behaviour of the spine. This is illustrated by the in vitro stiffness of the full-length caprine spine, which has been shown to reduce as muscle tissue is removed, in particular upon removal of the dorsal musculature [4]. Furthermore, in vitro biomechanical parameters differ considerably from in vivo conditions due to active neuromuscular control. As such, the behaviour of a biological system in its entirety (i.e. in vitro and in vivo) should be considered to allow a more comprehensive biomechanical evaluation of the suitability of an animal model for the human spine.

Range of motion of the human thoracolumbar spine in vivo has been investigated using a variety of measurement tools including kinematics, electromagnetic tracking devices, dynamometers, inclinometers, tape measure, and goniometry [5]. Although all methods are associated with some limitations [6], kinematic analysis using passive markers has previously been used for investigations of the human spine during active trunk movements [7]. In quadrupeds, kinematics of the thoracolumbar spine in vivo has also been reported using a similar approach in horses during induced active trunk movements [8] and during active cervical spine flexion exercises [9], however in vivo kinematics of the sheep spine during active exercises have not been quantified.

In man, surface electromyography (sEMG) is well established for the assessment of spinal muscle function, and it has been used to quantify muscle activation during a variety of conditions, examples of which include perturbations [10–12] and functional tasks [13–15]. Motor control and muscle coordination are also frequently investigated in man using sEMG [16–18]. Furthermore, the activation of spinal muscles during simple spinal range of motion exercises has been described [19,20]. Patterns of activation of the spinal muscles during similar active range of motion exercises have received very little attention in quadrupeds and it is limited to the muscles of the cervical spine in the horse [21]. To our knowledge, it has never been reported in sheep. In contrast, sEMG of spinal and trunk muscles has been used to investigate muscle activity during locomotion in sheep [22,23], dogs [24] and horses [25–30], thereby illustrating the suitability of sEMG for the investigation of trunk muscles in sheep during similar movements as those performed in human spinal investigations.

In order to better evaluate the relevance of in vitro spinal data obtained from sheep for extrapolation to the human spine, data on in vivo spinal biomechanics in sheep should be obtained using similar methodologies as those used in man. If considerable differences between human and sheep lumbar spine biomechanics were identified in vivo, despite the spinal biomechanics of these species being similar in vitro, the direct extrapolation of in vitro sheep data to the in vivo human spine, and thereby inferring likely outcomes of spinal interventions, should be questioned.

Furthermore, such inter-species comparisons should ideally be investigated across an age range of healthy sheep, to obtain basic spinal biomechanics data for comparison with the human spine at different ages, as reductions in muscle strength and functionality [31–35] and reductions in spinal range of motion [36–38] have been shown as a result of ageing in man. Therefore, between-species and between-age group differences in muscle activation should be considered.

Thus the purpose of the present study was to compare spinal range of motion and trunk muscle activation between healthy humans and sheep of different ages during active spinal range of motion exercises. It was hypothesised that spinal range of motion in sheep is smaller compared to that in man regardless of posture adopted. It was also hypothesised that significant differences in muscle activity would be found between humans and sheep, although these differences would be greatest when sheep were compared to humans adopting an upright bipedal stance.

## Materials and Methods

### Study population

Twenty-four healthy male and female participants were recruited. Inclusion criteria were age of 18–25 years (young group, n = 12, female n = 6) or 45–60 years (mature group, n = 12, female n = 6) and a Body Mass Index (BMI) of 18–25. Participants were excluded if they had current or a history of low back pain in the last 12 months, previous spinal surgery or fracture, neurological or orthopaedic disease, open abdominal surgery, or were not deemed eligible to undergo magnetic resonance imaging (MRI) as the present study was part of a larger study. All participants received an information sheet and gave written informed consent. Ethical approval for the humans was granted by the Medical University of Vienna Ethics Committee (1609/2012).

For the main ovine aspect of the study, 17 male and female Austrian Mountain sheep were purchased and allocated to an immature group (6–9 months n = 6, female n = 2), young group (1–3 years n = 6, female n = 6) or a mature group (6–9 years n = 5, female n = 2). In an additional aspect of the study, three further Austrian mountain sheep were included to determine the amount of skin displacement from radiography. Sheep were assessed by an experienced orthopaedic veterinarian and were deemed free from spinal or neurological disorders. Female sheep underwent an ultrasound investigation to exclude pregnancy. Ethical approval for the sheep was granted from the Austrian Federal Ministry of Science and Research (13/10/97/2011). All sheep were accustomed to wearing a halter and were trained to perform active spinal movements using food as an incentive.

### Measurement protocol

Age, height, and body mass were recorded for all humans. In the sheep, approximate age based on dentition for group allocation, height to the withers, and body mass were obtained.

**Fig 1:**
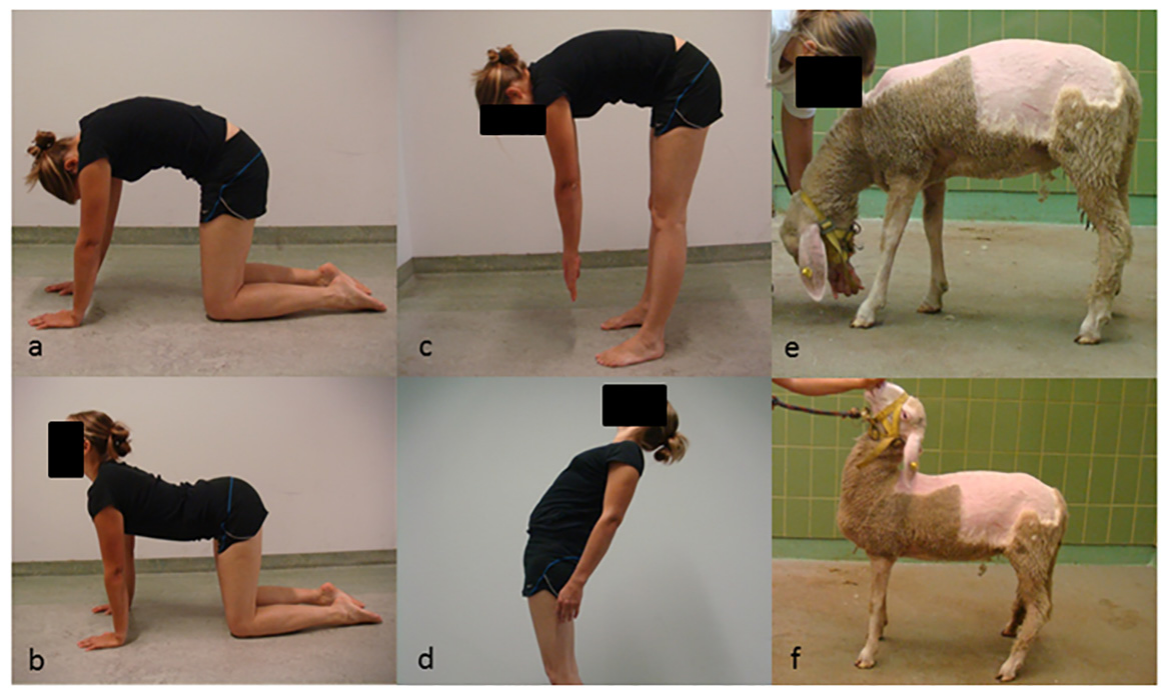
Active movements investigated: (a) human kneeling flexion (b) human kneeling extension (c) human standing flexion (d) human standing extension (e) sheep flexion (f) sheep extension.

Test movements were active spinal flexion and extension. Humans performed these movements in four-point kneeling (**Kn**eeling KN—hands and knees on the floor; Fig 1a and 1b) and in upright bipedal stance (**U**p**r**ight UR; Fig 1c and 1d). The individual in this manuscript has given written informed consent (as outlined in PLOS consent form) to publish these case details. Sheep performed these movements in their normal quadrupedal stance incentivised by food rewards (Fig 1e and 1f). Active movements were performed in a random order (3 trials per movement). Humans were instructed to perform each movement slowly as far as possible and return to the starting position, without maintaining end range of motion. In UR, human participants were asked to move the upper trunk backwards (for extension) or forwards (for flexion) over the pelvis. In KN, human participants were asked to arch their back up (for flexion) or hollow their back (for extension) as far as possible. Handlers used small amounts of feed to encourage the sheep to perform the movements slowly to their end range of motion and return to neutral, while avoiding the sheep taking a step to reach the food. Each individual movement trail was completed within 10 seconds for both humans and sheep.

### Data collection

**Fig 2:**
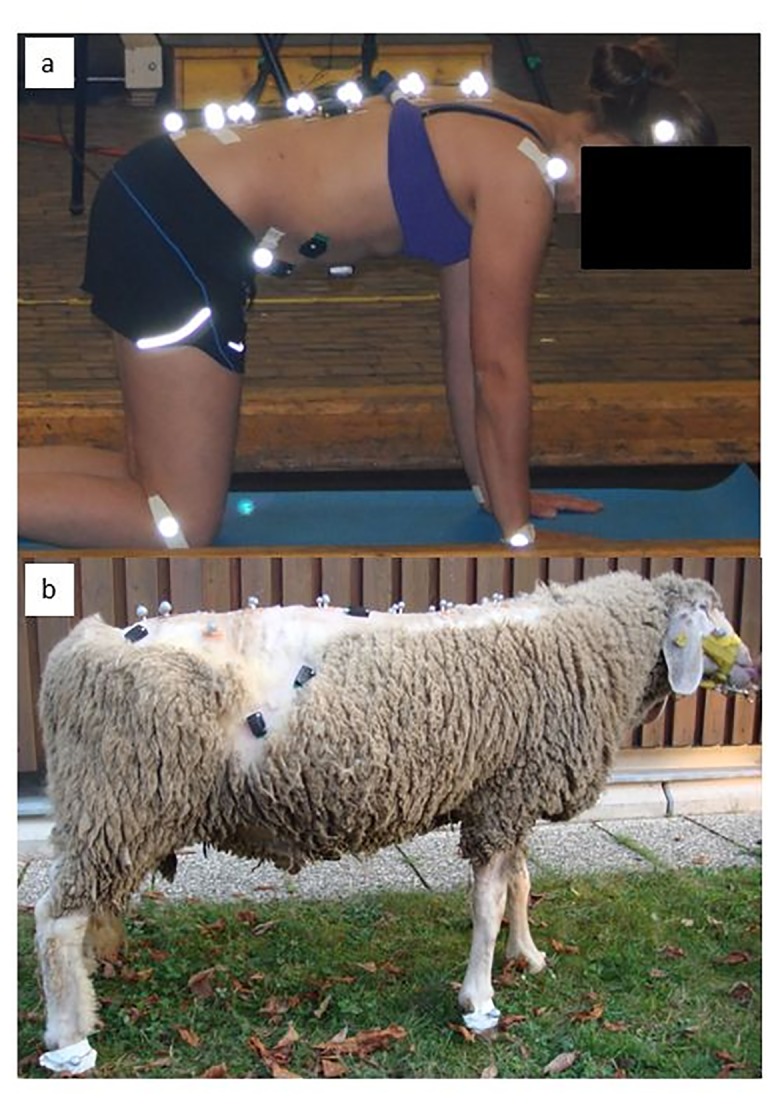
Human participant in four-point kneeling (a) and sheep (b), showing marker locations.

In humans, passive reflective markers were attached to the skin overlying the bony landmarks of the left and right ulnar styloid process, lateral femoral condyles, lateral malleoli, sacrum (S), left and right posterior superior iliac spines (PSIS), the spinous process and left and right transverse processes of the 5^th^ thoracic (T5), 8^th^ thoracic (T8), 11th thoracic (T11), 2^nd^ lumbar (L2) and 5^th^ lumbar (L5) vertebrae using adhesive tape, and three markers were attached to a headband worn by the participants (Fig 2a). In sheep, markers were attached laterally over all fetlock joints, sacrum (S), left and right tuber sacrale (TS), spinous process and left and right transverse processes of the fifth thoracic (T5), eighth thoracic (T8), 12^th^ thoracic (T12), third lumbar (L3), and seventh lumbar (L7) vertebrae, and three on the head (Fig 2b). A set of three spinal markers per anatomical location is referred to as triad. The slightly different vertebral locations between the species were chosen to reflect similar spinal levels, i.e. L5 (human) and L7 (sheep) being the last lumbar vertebra respectively. The sheep were shorn over the trunk to optimise marker visibility and a small drop of glue was used to ensure marker adhesion. An experienced musculoskeletal physiotherapist was responsible for marker placement in all humans, based on palpation of bony landmarks and an experienced orthopaedic veterinarian placed all spinal markers in the sheep. Latero-lateral radiographs (Computed Radiography, Imaging plate Fuji) of the thoracic and lumbar spine (70 kV, 2.1 mAs; Super 100 CP, Philips, Eindhoven, Netherlands) were obtained in a neutral spinal position in each sheep after spinous process markers were attached, to ensure marker placement was anatomically correct. In the additional three sheep used to determine skin displacement during active trunk motion, latero-lateral radiographs were also obtained with the thoracolumbar and sacrum markers attached whilst performing sustained end range flexion and extension.

In humans and sheep, three-dimensional kinematic data were collected using 10 infrared cameras (Eagle Digital Real Time System, Motion Analysis Corp., USA) with a sampling rate of 120Hz using kinematic software (Cortex 3.6.1).

In preparation for sEMG in humans, skin of was shaved where necessary, followed by gentle abrasion of the area with sand paper and cleaning with alcohol. The sheep were shorn, shaved, and the skin cleaned with alcohol followed by ether to degrease the skin. sEMG electrodes (Delsys Trigno, Boston, USA; parallel double bar electrodes 5mm x 10mm, gain 1000) were attached to the humans over the left and right sides of the following muscles: erector spinae (ES) 2 fingers width laterally from the thoraco-lumbar junction midline. The SENIAM [Surface Electromyography for the Non-Invasive Assessment of Muscles] guidelines recommends 2 fingers width laterally from the spinous process of the first lumbar vertebrae, but as contact loss was observed during extension in UR in pilot studies, the slightly more cranial location was selected), rectus abdominis (RA) 2cm above the umbilicus and 1cm lateral to midline [39], external obliquus (EO) midway between the ASIS and ribcage [40], and internal obliquus (IO) 2cm medially in a horizontal plane from the ASIS [39]. In the sheep, the comparable locations for sEMG electrode placements were selected for all muscles, as no guidelines or previous sEMG studies exist in sheep for the muscles investigated in the present study. Longissimus dorsi muscle, which is the ovine equivalent of the human ES, was used and referred to as ES in both species for clarity. In humans, sEMG electrodes were attached with non-irritant adhesive tape. In sheep, an additional small drop of glue was used to optimise electrode contact. Raw sEMG data were sampled at 1200Hz, passed through a 12 bit AD converter, and stored for processing. Kinematic and sEMG data were collected synchronously.

### Data analysis

**Fig 3:**
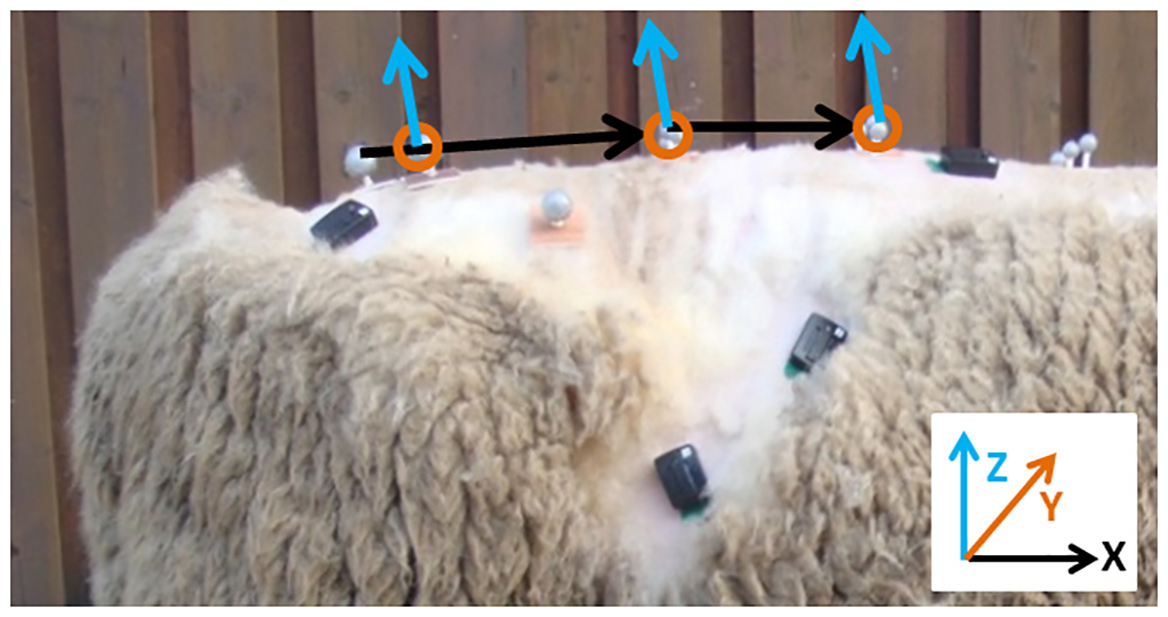
Vector orientations used to calculate spinal angles.

Kinematic data were visually inspected to ensure all movements were representative of the test movements and individual marker trajectories were smoothed with a low pass 6Hz Butterworth filter. Flexion and extension angles between triads were calculated with MATLAB (2012b), using a protocol similar to other studies [41–43]. In short (also see Fig 3), the x-axis for each spinal segment was defined i.e. in sheep from the sacrum or spinous process marker to the adjacent more cranially located spinous process marker (e.g. S and L7 spinous process, L7 and L3 spinous processes). The z-axis was defined from the cross product of the x-axis and a vector between the left and right transverse processes or left and right TS in sheep. The y-axis was defined as the cross product between the x and z axes. Vectors were normalised and a ZYX euler angle decomposition [42] was used to determine sagittal plane motion between adjacent vertebral segments. The resulting curves were smoothed using a moving average of 25 frames. Using this method, adjacent triads analysed were T8 and T11 in humans and T8 and T12 in sheep, T11 and L2 in humans and T12 and L3 in sheep, L2 and L5 in humans and L3 and L7 in sheep, and L5 and the sacrum in humans and L7 and the sacrum in sheep. Angles were expressed to the nearest degree. For each movement and posture, the mean value of the range of motion from the three measurement trials was taken. Data were excluded where either start or end range of motion was not available due to the markers being obscured from camera view or in sheep where steps were taken.

To determine the amount of skin displacement, the location of each marker relative to its spinous process was calculated using Sante Dicom viewer Freeware (v. 4.0.9) by defining the distance between two parallel lines: A line drawn from the centre of the kinematic marker (indicated by the tip of the screw visible) orientated perpendicularly to the dorsal aspect of the spinous process, and a second line drawn parallel to the first line which intersected either the most cranial or caudal aspect of the spinous process. The same reference point (cranial or caudal aspect of the particular spinous process) was used to calculate the amount of craniocaudal displacement of each kinematic marker between the end range flexion and end range extension position.

Raw sEMG signals were inspected for any obvious contact loss. Where this occurred, sEMG data from those muscles were excluded from those trials. Raw sEMG data were full-wave rectified, mean-offset, re-sampled to match kinematic data sampling and a 4^th^ order 6Hz low pass Butterworth filter was applied to obtain linear envelopes using scripts written in MATLAB (2008b). The largest (maximal) amplitude of each muscle (maximally observed sEMG [MOE]) was identified from each trial. From these maxima, the overall maximally observed sEMG (OMOE) was identified for each muscle and individual. MOEs were then normalised to the OMOE and presented as a percentage [MOE%], i.e. (MOE/ OMOE) * 100. The OMOE in sheep was identified from all flexion and extension trials, and in humans, the OMOE was identified from all flexion and extension trials in both UR and KN. The mean of the MOE%s was calculated for each movement and posture from all included trials.

The flexor-extensor difference (FED) was calculated from the mean MOE% values for each individual [44] for each movement and posture as follows:

$$\frac{\left(\text{RA left }+\text{ RA right }+\text{ OE left }+\text{ OE right }+\text{ OI left }+\text{ OI right}\right)}{6}-\frac{\left(\text{ES left }+\text{ ES right}\right)}{2}$$

The value in the denominator was amended if a muscle was excluded due to contact loss. Greater relative flexor muscle activity is indicated by a positive FED value, and a greater relative extensor muscle activity is indicated by a negative FED value.

Human sEMG data evaluation was conducted by one assessor, while in sheep, 10% of these files were selected at random and reviewed by a second assessor and data inclusion/exclusion was based on consensus opinion.

### Statistical Analysis

Normal distribution of the data was investigated using a Shapiro-Wilk test and by assessment of the frequency and Q-Q plots. Height and body mass were compared between the age groups for each species using an independent t-test (humans) and one-way ANOVA with Bonferroni post-hoc comparisons (sheep). A two-way factorial ANOVA with species and age group as variables was planned between sheep and UR humans, and between sheep and KN humans, but as there were not sufficient sheep kinematic data available for each age group and as sEMG data was not normally distributed, the age groups were combined. An independent t-test was used for the between-species comparison of spinal range of motion and a Mann-Whitney test was used for the between-species comparison of sEMG outcomes. Spinal range of motion was compared between human age groups using an independent t-test. Pearson’s correlation coefficient was used to investigate the association between spinal range of motion and age in sheep.

## Results

Mean age of the humans was 22.5 years (± 1.39) in the young group and 50.2 years (± 4.95) in the mature group. There were no significant differences for height and body mass between the human groups. Mean height and body mass of the combined groups of humans was 1.74m (± 0.11) and 68.85kg (±10.87). Range (mean ± standard deviation) of age, height and body mass of the combined age groups of sheep were 9 months to 8.5 years (3.85 ± 3.65), 0.66 to 0.98m (0.82 ± 0.12) and 41.5 to 113.5 kg (69.60 ±28.22). Mean age and body mass of the three additional male sheep used to determine skin displacement were age 4.3 ± 0.81 years, body mass 105.5 ± 9.93kg.

**Fig 4:**
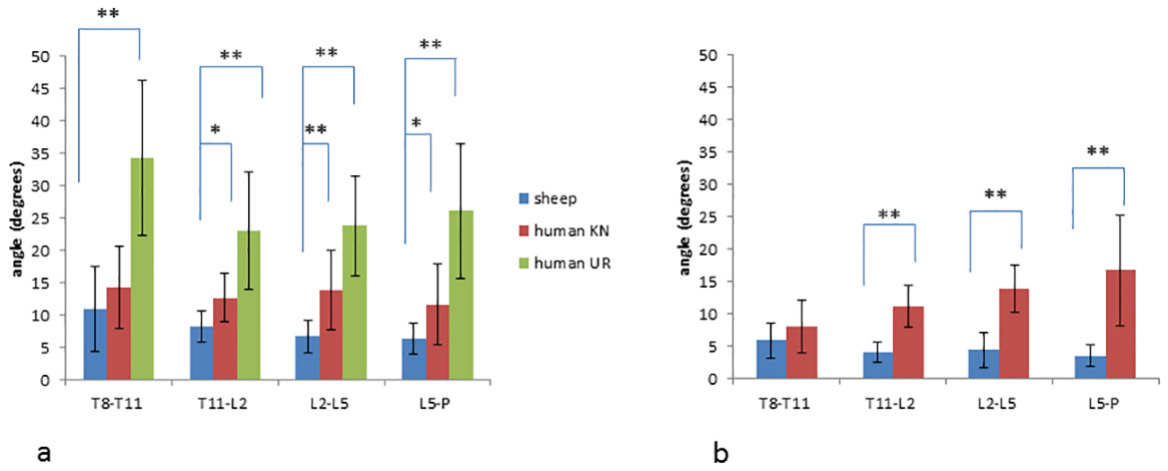
Mean (standard deviation as error bars) of range of motion in degrees between adjacent vertebral segments during flexion (a) and extension (b) in sheep and human participants in an upright (UR) and four-point kneeling posture (KN). Brackets and asterisks indicate significance differences (*p<0.05, **p<0.001) between humans and sheep.

The mean ranges of motion between adjacent triads for each species, movement direction and posture are shown in Fig 4. As all trials of humans led to a loss of marker visibility during UR extension, no data are available for this movement and posture. For the comparison between sheep and KN humans, all adjacent triads except T8-T11/T12 showed a significantly smaller range of motion in the sheep during both flexion (all p<0.05) and extension (all p<0.001). For the comparison of flexion between sheep and UR humans, sheep had a significantly smaller range of motion between all adjacent levels (all p<0.001). There were no significant differences in spinal range of motion between the young and the mature humans, and there were no significant correlations between sheep spinal range of motion and age.

**Fig 5:**
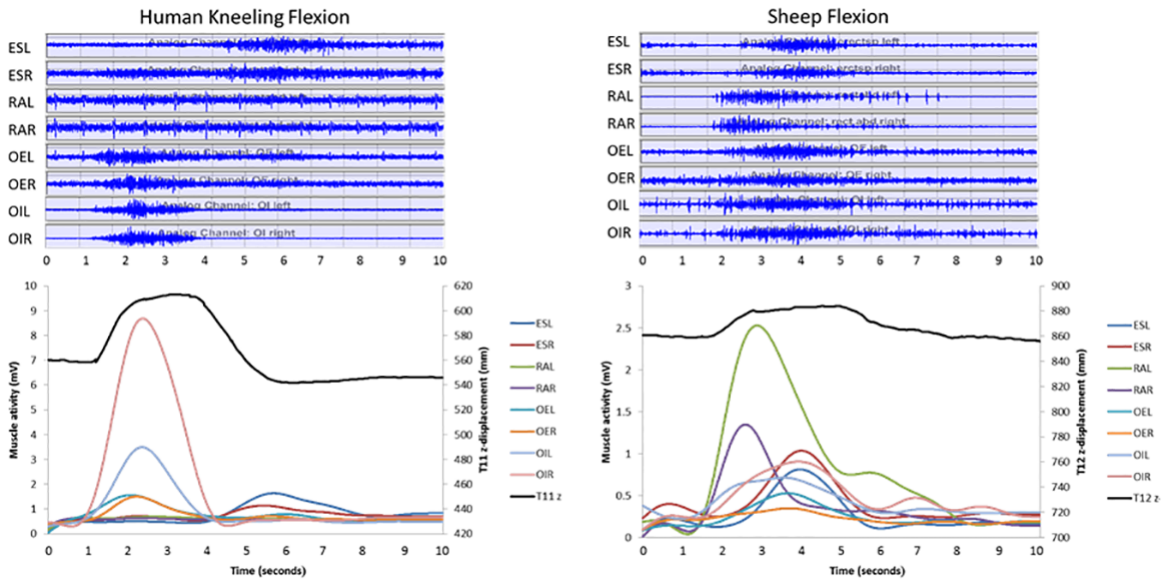
Examples of raw (top images) and filtered (lower images) EMG signals of the muscles erector spinae left (ESL), erector spinae right (ESR), rectus abdominis left (RAL), rectus abdominis right (RAR), obliquus externus left (OEL), obliquus externus right (OER), obliquus internus left (OIL), and obliquus internus right (OIR) with accompanying z-displacement of the 11^th^ (human) or 12^th^ (sheep) thoracic marker for flexion in a mature human participant in kneeling (left), and a mature sheep (right).

Examples of human and sheep raw and processed sEMGs are shown in Fig 5. MOE% values during flexion and extension are displayed in Table 1. During extension, MOE% of ES was greater in sheep compared to UR humans (p<0.001), MOE% of OE and OI was greater in sheep compared to KN humans (p<0.001), and MOE% of RA was smaller in sheep compared to UR humans (p<0.01). During flexion, MOE% of ES was greater in sheep compared to KN humans (p<0.001) but smaller compared to UR humans (p<0.05), while MOE% of RA was greater in sheep compared to both KN and UR humans (p<0.01). Unilateral differences were found for the MOE% of left OE which was greater in sheep compared to KN humans (p = 0.003), and for MOE% of right OI, which was greater in sheep compared to UR humans (p = 0.004).

**Table 1:** Medians of the muscle activity of erector spinae left (ESL), erector spinae right (ESR), rectus abdominis left (RAL), rectus abdominis right (RAR), obliquus externus left (OEL), obliquus externus right (OER), obliquus internus left (OIL), and obliquus internus right (OIR) as a percentage of the maximally observed EMG (%MOE) in humans in kneeling (KN) and upright (UR) postures and in sheep for extension (E) and flexion (F) movement. P-values indicate between species significant differences for human kneel vs sheep, and human upright vs sheep conditions. Non-significance is indicated by NS.

EXTENSION	Muscle	Posture	human	sheep	p-value	FLEXION	Muscle	Posture	human	sheep	p-value
	ESL	KN	86.78	80.76	0.352		ESL	KN	14.35	51.71	<0.001
		UR	22.77		<0.001			UR	71.21		0.030
	ESR	KN	86.62	73.98	0.601		ESR	KN	14.54	39.75	<0.001
		UR	27.87		<0.001			UR	73.51		0.001
	RAL	KN	17.36	24.24	0.934		RAL	KN	48.67	80.80	0.018
		UR	80.97		0.002			UR	39.05		0.002
	RAR	KN	16.79	19.18	0.987		RAR	KN	46.17	85.37	0.001
		UR	84.41		<0.001			UR	43.71		0.004
	OEL	KN	19.68	79.82	<0.001		OEL	KN	30.55	68.95	0.003
		UR	77.92		0.463			UR	55.29		0.345
	OER	KN	23.21	72.57	<0.001		OER	KN	42.95	65.07	0.172
		UR	82.04		0.296			UR	52.78		0.521
	OIL	KN	20.63	72.85	<0.001		OIL	KN	74.61	74.30	0.777
		UR	79.09		0.823			UR	47.96		0.061
	OIR	KN	18.05	61.77	<0.001		OIR	KN	55.19	74.15	0.368
		UR	80.96		0.432			UR	47.72		0.004

FED was significantly different between sheep and humans in both an UR and KN postures in both movement directions (see Table 2). For flexion, sheep used significantly less abdominal activity compared to humans KN (p = 0.044), but human UR used significantly more extensor muscle activity compared to sheep (p<0.001). For extension, human KN required more extensor muscle activity than sheep (p<0.001), but humans UR used more abdominal muscle activity compared to sheep (p<0.001).

**Table 2:** Medians with lower and upper quartiles in parentheses of the Flexion-Extension Difference (FED) between the abdominal muscles and spinal extensor in sheep and in humans in a kneeling (KN) and upright (UR) posture during flexion and extension. A positive value indicates greater abdominal activity. Identical superscripts indicate significant differences between sheep and human conditions.

	Flexion	Extension
Sheep	19.02 (8.73, 33.47)^a^^,^^b^	-19.39 (-30.62, -12.32)^c^^,^^d^
Human KN	31.52 (18.29, 49.950)^a^	-60.76 (-69.87, -39.46)^c^
Human UR	-23.85 (-41.12, 2.41)^b^	43.81 (25.14, 62.51)^d^

p-value:

^a^ = 0.044

^b^ < 0.001

^c^ < 0.001

^d^ < 0.001

Skin displacement of the markers overlying the spinous processes of T5, T8, T12, L3, L7 and the sacrum from end range spinal flexion to end range spinal extension in the additional three sheep are shown in Table 3. Due to difficulties in the accurate interpretation of the thoracic images for the marker at T5 in two sheep, the value for this marker in the table represents only one sheep, whereas all other markers represent data from all three sheep.

**Table 3:** Mean and standard deviation (stdev) of skin displacement in mm of the markers overlying the sacrum and the spinous processes of L5, L2, T12, T8, and T5. Data from all three sheep is presented except for the marker over T12, which is only available from one sheet.

Spinal location	Skin displacement (mm)	
	Mean	stdev
sacrum	9.60	6.10
L5	15.22	4.00
L2	30.30	7.64
T12	35.37	
T8	32.07	5.38
T5	86.82	14.00

## Discussion

This is the first in vivo study to report and compare spinal range of motion and muscle activity between humans and sheep, a commonly used animal model for the human lumbar spine. As hypothesised, sheep spinal range of motion was significantly smaller compared to that of man adopting a normal bipedal stance and a four point kneeling (quasi-quadrupedal) posture. Furthermore, distinct differences in the maxima of the normalised sEMGs between humans and sheep were found.

The moderately high activity of the oblique abdominal muscles in sheep regardless of movement direction is thought to be required to control the relatively large abdomen with the voluminous rumen; such an abdomen is absent in healthy weight humans and therefore this does not pose the same biomechanical challenges. During flexion in sheep, RA appeared to be the main agonist. This was less apparent in humans where the synchronous activity of RA with the other abdominal muscles during flexion indicates that the work of flexion is shared between the abdominal muscles. Alternatively, it may be that RA requires relatively greater work to create a flexion moment to counteract the effects of gravity on the sheep abdomen. It has been proposed that RA is particularly important in quadrupeds due to its long lever arm and close association to the *linea alba*, which being a passive tensile band, offers support to the trunk and abdomen [45].

Another factor to be considered is the effective stiffness of the human and ovine spine. In vitro stiffness of the thoracolumbar spine has been described in sheep [2] and in the humans [46], although methodological differences between laboratories make direct comparison limited. Stiffness of the cervical spine however has been directly compared between humans and sheep in the same laboratory using the same test set-up, which showed greater stiffness in the human specimens for the cranial cervical spine, but greater stiffness in the sheep specimens in the caudal cervical spine [47]. Also between quadrupedal species, different stiffness values have been reported for the thoracolumbar spine when similar test set-ups were used. For example, reported caudal thoracic adult sheep spine stiffness [2] is lower than that described in the caudal thoracic spines in calves [48]. The higher agonistic activity of RA during flexion in sheep could therefore be indicative of the sheep spine being stiffer than the human spine, as it could be suggested that a stiffer spine requires greater muscle work to create spinal motion. However, muscle activity of ES was not found to be greater in sheep during extension, probably as the mass of the abdomen is assisted by gravity and relaxation of the abdominal muscles is sufficient to allow spinal extension.

When comparing the two postures adopted by the humans in the present study (UR and KN) to the sheep, the KN position with its smaller range of spinal motion in flexion was more similar to the ovine flexion. This is likely to be due to both lower/pelvic and upper / thoracic limbs being in a weight-bearing position, which reduces the freedom of motion of the upper trunk and thus the ability of the thoracolumbar spine to move through its full available range is reduced. However, sheep spinal range of motion was still significantly smaller compared to KN humans, indicating that quadrupedal posture alone is not responsible for the smaller range of motion observed in the sheep thoracolumbar spine. Several reasons can be hypothesised. The differences in spinal range of motion between humans and sheep may be due to a combination of differences in skeletal anatomy i.e. ratio of vertebral body height to width, and anterior disc heights [1], and in spinal alignment (i.e. lumbar lordosis in humans and lumbar kyphosis in sheep). Furthermore, sheep very rarely rear up or perform other tasks involving bipedal support and the quadrupedal posture is their standard body posture. Although humans adopt a range of postures in activities of daily living, these are primarily restricted to an upright / bipedal posture, and kneeling postures are less common in adults. Therefore, the musculoskeletal system of humans has not adapted to be efficient in a quadrupedal posture, whereas it is assumed that sheep have evolved a spine with a smaller range of motion to suit their quadrupedal posture. Without this, the passive structures including the long and short spinal ligaments and the abdominal muscle forces might not be sufficient to stabilise against gravitational extension of the thoracolumbar spine in quadrupeds, or the energetic cost simply be too great. These factors may also explain why humans in a kneeling posture used more abdominal muscle activity for a flexion movement and more extensor muscle activity for an extension movement compared to sheep (based on FED values); the novelty of the exercise may have required the humans to use relatively greater agonistic activity to perform the tasks and they had a greater range of motion available to do so. One further observation which was not quantified in the present study was that the cervical region contributed a considerable amount of motion to overall motion in the sheep. It may be that the relative motion of different anatomical regions of the spine varies between humans and sheep, i.e. greater in the cervical region and less in the lumbar region in sheep compared to man. This would benefit from further investigation in future studies.

As yet, no other studies investigating in vivo sheep spinal range of motion are available for comparison. However, in vertebral specimens of 4-year old Merino sheep with muscles removed but intervertebral ligaments kept intact, flexion-extension range of motion of single-joint segments in the thoracic spine was reported to range from 2 to 5°, whereas in the lumbar spine these values ranged from 4 to 6° [2]. This is similar to the human lumbar spine in vitro devoid of musculature but intervertebral ligaments kept intact as median range of motion of combined flexion-extension of L4-L5 was reported as 6.5° in adult L2-Sacrum specimens [49]. From all spinal angles measured in sheep in the present study, combined flexion and extension range of motion ranged from 8° to 17° and after correction for the number of vertebrae present between the marker triads, these values equate to 3 to 8° per thoracolumbar spine motion segment, which is similar to the motion of single-joint segments previously found by Wilke *et al* [2]. The combined flexion-extension range of motion of the lower lumbar spine in KN humans in the present study (28° spanning three intervertebral joints) may be slightly greater than in vitro L4-L5 motion reported by Wilke *et al* [49] after correcting for the number of motion segments between the marker triads, despite a similar age range of cadavers (28 to 63 years) used in the study by Wilke *et al* [49] and participants used in the present study.

There are few reports of spinal range of motion in humans adopting a kneeling posture, and to our knowledge there are none which have used a detailed kinematic marker set-up allowing 3D angle calculations. However, using a basic marker set-up to determine a 2D spinal angle during flexion performed in UR and KN, Kuo *et al* [6] showed a significantly greater flexion range of motion in UR compared to KN. In the present study, age-related changes in spinal behaviour were investigated as a potential confounding factor on the study results, and no differences between age groups were identified; this is in contrast to Kuo *et al* [6], where a significantly smaller thoracic spine range of motion was found in the mature group of 60–83 years (34 ± 16°) compared to a young group of 17–27 years (49 ± 12°). As the mature group in the present study was considerably younger than the mature group in the study of Kuo *et al* [6], the lack of age effects in spinal range of motion in the present study is probably explained by the smaller difference between the age groups.

Many spinal research studies use young, skeletally mature sheep [50] or report sheep as being skeletally mature without more detail to actual ages being provided [51–53]. Neither differences in spinal range of motion between the humans age groups, nor significant correlations between age and range of motion in sheep were not found in the present study. However, as ageing has been shown to influence spinal motion in other studies [6,35–37], future investigations evaluating spinal interventions destined to be used in an older human population should endeavour to use appropriately age-matched sheep. As growth of the spines of Swiss alpine sheep was shown to be ceased by 15 to 18 month of age [54], it is unlikely that young sheep will have degenerative changes similar to man for which interventions are being sought, as such changes occur in sheep from the age of 6 years in the ovine intervertebral disc [55].

Active spinal movements similar to those performed in human studies (i.e. feet remaining stationary and the spine flexed or extended) are less frequently investigated in quadrupeds, and to our knowledge this is limited to the equine species, where a first description of spinal range of motion during induced movements reported spinal displacement [8]. In a more recent study which used a similar set-up as that used the present study, flexion range of motion of two vertebral junctions in the equine lumbar spine was found to range from 1 to 5°, while in the caudal thoracic spine a greater amount of flexion (6 to 7°) was found [9]. Although these values are greater than the flexion range of motion observed in the sheep in the present study, a similar regional trend of greater range of motion in the thoracic spine in comparison to the lumbar spine was seen.

There are limitations in the present study which should be acknowledged. Kinematic analysis of the thoracolumbar spine using a kinematic marker set-up similar to the present study has been applied in man previously. For example, Wade *et al* [43] investigated lumbar spine angles in elite female gymnasts during a landing task, and Ebert *et al* [41] evaluated side flexion motion at end range spinal flexion and extension; these studies indicate the feasibility of this marker set-up. However, neither of these studies investigated full thoracolumbar extension similar to that performed in the present study. For example, the participants in the study by Ebert *et al* [41] performed an anterior pelvic tilt with a lordosis in both sitting in standing, whereas in the present study, participants were asked to perform full thoracolumbar extension, moving the upper trunk backwards over the pelvis. Although this will lead to a more natural and representative movement pattern, it caused markers to be obstructed from camera view, and for this reason it was not possible to report extension range of motion in standing humans. Camera positioning from the floor up would have been required, but this set-up would have been unsuitable for the data collection of both UR flexion and KN flexion and extension, and the logistics of the data acquisition prevented us from using two camera set-ups one after the other. Kinematics of full range, standing human thoracolumbar extension has not been reported previously to our knowledge, probably for this reason. Inertial sensors, which do not rely on a camera system, could be beneficial for similar extension studies in the future.

Skin movement is a known limitation in kinematic and sEMG studies, and inter-individual differences in skin displacement in man have been reported [56]. This limitation has also been described in a canine sEMG study, where relatively loose skin in some dog breeds may have led to increased cross-talk in the sEMG signal [24]. The use of bone pins for kinematic markers eliminates the potential error of skin displacement, and this method has been used to quantify motion in the human spine during locomotion [57,58]. However, bone pins cannot be readily applied due to their invasive nature. Furthermore, bone pins will also influence the movements investigated due to possible discomfort or skin tethering on the bone pins, particularly in large dynamic ranges of motion. The influence of bone pins onto sEMG recordings in the vicinity is as yet un-documented, but a change in muscle activity has to be expected with such a method. In order to quantify skin displacement in sheep, which has not previously been reported, craniocaudal skin displacement in three large Austrian mountain sheep was determined in the present study based on radiographs in maximum flexion and extension, similar to the movements investigated with kinematics and sEMG. These data are thought to present the maximal likely skin displacement observed in the main study as the sheep used to determine skin displacement were larger than the sheep used in the main study. Skin fixated markers have shown good agreement with the location of spinal landmarks based on open MRI evaluation in man during flexed postures, with skin displacement of up to 25mm over the lumbar spine reported during maximal spinal flexion [59]. These values are similar to the skin displacement over the lumbar spine determined in the sheep in the present study.

Normalisation of EMG signals is recommended to minimise the amount in inter-and intra-variability in EMG signals known to occur in human subjects [17,60], and this is also assumed to occur in sheep. Due to the additional variability caused by the interspecies comparison in the present study (i.e. potential differences in skin thickness, impedance, etc.), applying a normalisation technique is imperative to allow comparisons to be made. In man, isometric maximum voluntary contraction (MVC) is most frequently used as a means of EMG normalisation, including the trunk muscles and spinal extensors. Unfortunately, this method is not feasible in animal studies; nor for that matter is it suitable in humans with potential spinal pain. As the sheep are used to test spinal interventions used in a clinical (human) population, using a method of sEMG normalisation which is suitable to both an animal and patient population further supports the appropriateness of a non-MVC method of sEMG normalisation. In addition, although there are several benefits of the MVC method of normalisation when isometric conditions are investigated, the usefulness of this approach for dynamic tasks has been questioned and mean or peak dynamic normalisation is discussed to be potentially more suitable for normalising EMGs obtained from dynamic activities [60,61]. For this reason, in the present study normalisation was performed based on the maximally observed EMG, which has also been used in other animal EMG studies [21,23].

All sheep muscles investigated in the present study showed acceptable sEMG traces in the majority of measurement trials, despite there being no recommendations for sEMG electrode placement in sheep and thus locations comparable to the human locations were used to allow a between-species comparison. Furthermore, at the time of data collection, selection of the sEMG location for the abdominal muscles in man was based on locations used in previously published studies. More recently, new recommendations for sEMG sensor placement for OI in man have been published, this being 2cm below the most prominent part of the anterior superior iliac spine [62] and different from the location used in the present study, which was slightly further cranial. This may have influenced the traces of this muscle and therefore this difference in location should be taken into consideration when comparing the results of the present study with future studies.

In conclusion, significant differences in spinal range of motion and trunk muscle activity between sheep and man exist. Even though these differences are overall smaller when comparing sheep with KN humans rather than UR humans, they remain of a magnitude that shows anatomical and functional differences which go beyond postural effects. The findings of the present study thus illustrate the influence of in-vivo parameters to be considered before ovine model data is directly translated to man. In vitro spinal range of motion in man and sheep may be similar and the quadrupedal spine is thought to experience similar axial loads compared to man, however the smaller range of spinal motion in sheep in vivo may suggest that spinal implants tested in a sheep model may experience different stresses compared to those experienced in man. Although speculative at this stage, this could potentially mask the type or rate of development of implant failures, particularly in the long-term after repeated loading, and this warrants further investigation. As a future perspective, the exploration of muscle function in other quadrupeds and its comparison against man will enhance the applicability of the results of such in vivo interspecies comparisons.
